# A52 THE LGR5 ACTIVITY MODULATES CELL METABOLISM TO FAVOR WOUND HEALING

**DOI:** 10.1093/jcag/gwad061.052

**Published:** 2024-02-14

**Authors:** J A Acosta Montalvo, G Arguin, S Dagenais Bellefeuille, F Gendron

**Affiliations:** Immunology and cell biology, Universite de Sherbrooke, Sherbrooke, QC, Canada; Immunology and cell biology, Universite de Sherbrooke, Sherbrooke, QC, Canada; Immunology and cell biology, Universite de Sherbrooke, Sherbrooke, QC, Canada; Immunology and cell biology, Universite de Sherbrooke, Sherbrooke, QC, Canada

## Abstract

**Background:**

The stem cell marker leucine-rich G-protein-coupled receptor-5 (LGR5) acts as an R-spondin (RSPO) signal transducer, facilitating WNT signaling to support tissue development and renewal. In GI diseases, LGR5^+^intestinal stem cells play a critical role in wound healing, while its expression by cancer stem cells correlates with cancer progression and chemoresistance. Surprisingly, only a handful of studies have investigated the functional role of LGR5 as a receptor. Demonstrating LGR5 receptor function and identifying signaling determinants will be a significant breakthrough in understanding stem cell biology under normal and pathological conditions. The project hypothesis is that LGR5 scaffolds the assembly of signaling complexes that modulate cell metabolism to accommodate cell fate, growth, and migration.

**Aims:**

The project aims to characterize the molecular determinants linking LGR5 to cell metabolism and wound healing.

**Methods:**

As a working model, recombinant LGR5 and mutant constructs were expressed in HEK293 cells. Wound healing was determined using scratch and single-cell tracking assays to measure cell migration. Cellular metabolism was measured using Seahorse assays complemented by metabolomic studies.

**Results:**

Proteomic analysis revealed a rich LGR5 protein interactome associated with amino acid, glucose, purine metabolism, scaffold proteins (*e.g.,* 14-3-3), and GPCR signaling. Using Seahorse assays, we showed that oxygen consumption and glycolysis were increased in LGR5-expressing cells, while metabolic profiling revealed enrichment in metabolites associated with glutamate, purine, pyrimidine metabolism, pentose phosphate pathway, glycolysis, and TCA cycle, among others. Finally, LGR5 expression promoted wound healing and a single cell's migration distance.

**Conclusions:**

The results describe for the first time a functional role of LGR5 as a receptor, but foremost as an effector modulating cell metabolism. The findings may pave the way for new therapeutics targeting the LGR5 interactome to restore tissue homeostasis in diseases such as inflammatory bowel disease or to block cell adaptation and dissemination in the context of cancer.

**Funding Agencies:**

CCCNSERC

